# Nectar cardenolides and floral volatiles mediate a specialized wasp pollination system

**DOI:** 10.1242/jeb.246156

**Published:** 2024-01-04

**Authors:** Hannah Burger, Samantha Buttala, Hauke Koch, Manfred Ayasse, Steven D. Johnson, Philip C. Stevenson

**Affiliations:** ^1^ Evolutionary Ecology and Conservation Genomics, Ulm University, 89081 Ulm, Germany; ^2^ Royal Botanic Gardens, Kew, Kew Green, Richmond TW9 3AE, UK; ^3^ Centre for Functional Biodiversity, School of Life Sciences, University of KwaZulu-Natal, Scottsville, Pietermaritzburg 3209, South Africa; ^4^ Natural Resources Institute, University of Greenwich, Chatham Maritime, Kent ME4 4TB, UK

**Keywords:** Toxins, Wasp flower, Nectar chemistry, Floral scent, Acetic acid, Apocynaceae

## Abstract

Specialization in plant pollination systems can arise from traits that function as filters of flower visitors. This may involve chemical traits such as floral volatiles that selectively attract favoured visitors and non-volatile nectar constituents that selectively deter disfavoured visitors through taste or longer-term toxic effects or both. We explored the functions of floral chemical traits in the African milkweed *Gomphocarpus physocarpus*, which is pollinated almost exclusively by vespid wasps, despite having nectar that is highly accessible to other insects such as honeybees. We demonstrated that the nectar of wasp-pollinated *G. physocarpus* contains cardenolides that had greater toxic effects on *Apis mellifera* honeybees than on *Vespula germanica* wasps, and also reduced feeding rates by honeybees. Behavioural experiments using natural compositions of nectar compounds showed that these interactions are mediated by non-volatile nectar chemistry. We also identified volatile compounds with acetic acid as a main component in the floral scent of *G. physocarpus* that elicited electrophysiological responses in wasp antennae. Mixtures of these compounds were behaviourally effective for attraction of *V. germanica* wasps. The results show the importance of both volatile and non-volatile chemical traits as filters that lead to specialization in plant pollination systems.

## Teaser

**Highlighted Article:** A combination of floral volatiles and nectar chemicals mediate plant–pollinator interactions. The nectar of wasp-pollinated *Gomphocarpus physocarpus* contains cardenolides functioning as a filter against disfavoured visitors.

## INTRODUCTION

Many flowering plants have morphological and chemical filtering mechanisms that restrict nectar access to flower visitors that provide the most effective pollination service (Shuttleworth and Johnson, 2009a; Willmer, 2011; Dellinger, 2020; van der Kooi and Ollerton, 2020). These floral adaptations can contribute to the development of specialized pollination systems (exclusive pollination by particular functional pollinator groups), which can increase pollination efficiency (Fenster et al., 2004). A key function of floral filters is to limit floral visitors that feed on floral rewards, but do not pollinate the flowers effectively because they do not contact reproductive organs (Irwin et al., 2010).

Nectar is typically deployed by plants as an energy reward to attract and retain pollinators (Nicolson and Thornburg, 2007). However, nectar also contains a wide range of plant secondary metabolites (Palmer–Young et al., 2019), some of which also serve as defence compounds elsewhere in the plant and thus tend to be toxic to flower visitors (Stevenson, 2020). These secondary metabolites can act as floral filters that deter some flower visitors, but not others. In *Rhododendron simsii*, for example, the diterpenoid grayanotoxin occurs in nectar at concentrations that are toxic to honeybees, but not to *Bombus terrestris* bees (Tiedeken et al., 2016). Similar cases of a function for nectar secondary metabolites in filtering out less-desirable flowers visitors have been reported for *Aconitum* (Barlow et al., 2017) and *Aloe* (Johnson et al., 2006).

Filtering of floral visitors by morphology and nectar chemistry is not the only basis of specialization in pollination systems. Flower colour and volatile emissions can play an important role in selective attraction of particular floral visitors (Schiestl and Johnson, 2013; Scott-Brown et al., 2019). These traits are important not only for initial location of floral host plants by pollinators based on innate responses, but also for subsequent associative conditioning (Raguso, 2008; Burger et al., 2013; Schiestl and Johnson, 2013).

Many plant species are pollinated exclusively by wasps (Weiblen, 2002; Shuttleworth and Johnson, 2012), but the basis of this specialization is still poorly understood, with the exception of examples where flowers mimic female wasps (Schiestl et al., 2003) or their prey (Brodmann et al., 2008, 2009). Even the role of volatiles in the well-known fig wasp system is still not fully resolved (Chen et al., 2009). Flowers pollinated by wasps are often drab coloured and experiments have shown that visual cues are often not a requirement for wasps to locate flowers (Shuttleworth and Johnson, 2009b). Instead, specialization in flowers pollinated by nectar-seeking wasps may be based on a combination of volatile signals and chemical filters in nectar (Shuttleworth and Johnson, 2012; Burger et al., 2017). However, both the active volatile scent compounds that attract wasps and the non-volatile nectar compounds that repel other visitors, such as honeybees, have not yet been identified in these systems.

Wasp pollination is particularly developed in African milkweeds (Shuttleworth and Johnson, 2006, 2008; Burger et al., 2017). For example, the flowers of the milkweed *Gomphocarpus physocarpus* are pollinated almost exclusively by vespid wasps in both its native range in Africa and the invasive range (Coombs et al., 2009; Ward and Johnson, 2013; Burger et al., 2017). Although the flowers have no morphological barriers limiting access to the openly presented nectar, the flowers are only occasionally visited by honeybees (Coombs et al., 2009) that are otherwise abundant visitors to flowers of the related species *G. fructicosus* (Burger et al., 2017). Milkweeds (Apocynaceae: Asclepiadoideae) produce cardenolides that act as effective defensive compounds to reduce herbivore damage (Agrawal et al., 2012). Cardenolides are known to occur in the genus *Gomphocarpus* (Groeneveld et al., 1990), but their occurrence in *G. physocarpus* and *G. fruticosus* and their effect on floral visitors is unknown. The toxins inhibit animal Na^+^/K^+^-ATPase, but some insects have evolved strategies to tolerate these chemicals (Agrawal et al., 2012). Monarch butterflies, for example, sequester cardenolides from their milkweed host plants in the larval stages as a defence against predation (Brower et al., 1968; Dobler et al., 2012).

The nectar of many milkweeds contains a suite of putatively toxic cardenolides at a range of concentrations depending on the species (Manson et al., 2012; Villalona et al., 2020), but their effect on nectar-seeking visitors is still poorly studied. The toxic effects of cardenolides are often compound-specific (Detzel and Wink, 1993) and they differ in their toxicity, distastefulness and rate of post-consumptive effects (Malcolm and Brower, 1989) and show possibly synergistic effects. However, up to now, behavioural experiments were only performed with single commercially available cardenolides that do not naturally occur in the studied systems (Villalona et al., 2020). Nectar foraging monarch butterflies, which are not effective pollinators of milkweeds (Jennersten and Morse, 1991; Kephart and Theiss, 2004), are not deterred by the cardenolide ouabin (Jones and Agrawal, 2016). *Bombus impatiens* bumblebees did not avoid digoxin (Manson et al., 2012), and avoided ouabain only after extended foraging periods (Jones and Agrawal, 2016). In contrast, a more specialized bee visitor of milkweeds, *B. griseocollis*, showed an increased ability to both detect and tolerate this cardenolide (Villalona et al., 2020).

The aim of this study was to test the hypothesis that wasp pollination of *G. physocarpus* is mediated by nectar chemistry and the floral volatiles that attract the wasps, but deter other pollinators including honeybees. We hypothesized that nectar cardenolides of *G. physocarpus* are toxic to honeybees but do not negatively affect wasps. Olfactory cues of *G. physocarpus* flowers are highly attractive for wasp pollinators (Burger et al., 2017), but the biologically active scent constituents have not yet been identified.

We undertook a series of experiments to address the following research questions. Does nectar of the wasp-pollinated *G. physocarpus* and bee-pollinated *G. fruticosus* differ chemically, particularly with respect to the structures and concentrations of cardenolides? Do *Apis mellifera* honeybees incur negative effects such as mortality or deterrence when feeding on nectar of *G. physocarpus*? Do honeybees and *Vespula germanica* wasps differ in consumption and mortality of individuals when feeding on cardenolide fractions of *G. physocarpus* and *G. fruticosus* in different concentrations? Do antennae of *A. mellifera* bees and *V. germanica* wasps respond to specific compounds in the floral scent of *Gomphocarpus* spp.? Do active volatile compounds identified in *G. physocarpus* flowers attract *V. germanica* wasps?

## MATERIALS AND METHODS

### Study species

**Fig. 1.**
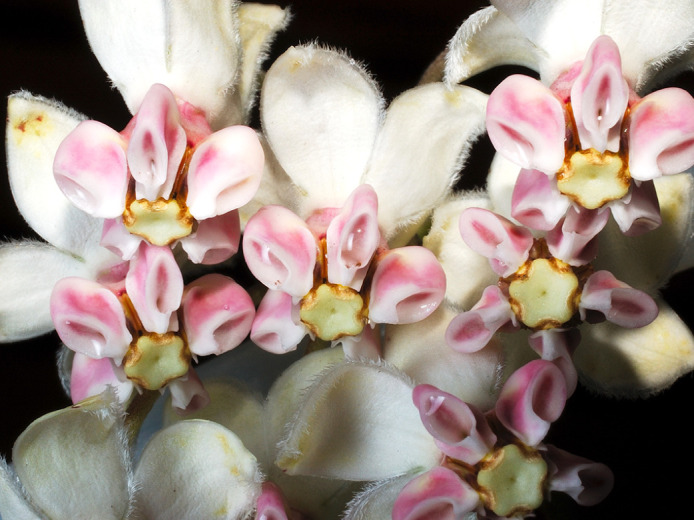
Inflorescence of *Gomphocarpus physocarpus* with nectar accumulated in corona lobes of individual flowers.

*Gomphocarpus physocarpus* and *Gomphocarpus fruticosus* (Apocynaceae: Asclepiadeae) are native to South Africa, but are now distributed worldwide in regions with adequate climatic conditions, e.g. the Mediterranean, Australia and Hawaii (Ward et al., 2012; Palma et al., 2023). The distribution range of the two species partly overlaps in the native range (Goyder and Nicholas, 2001; Coombs et al., 2009). *Gomphocarpus physocarpus* is mainly pollinated by vespid wasps in its native and non-native range (Forster, 1994; Coombs et al., 2009; Ward and Johnson, 2013). The pollination system is described to be specialized at the level of functional group (medium-sized vespid wasps), but generalized across different species of Vespidae (Coombs et al., 2009). The flowers have an open morphology and offer copious amounts of nectar (Fig. 1) so that the wasps can easily reach the nectar with their relatively short glossae (Burger et al., 2017). In contrast, *G. fruticosus* is mainly pollinated by bees, with honeybees among the most frequent visitors in its native (Burger et al., 2017) and wider distribution range (Coleman, 1937). Beside bees, *G. fruticosus* is also visited by other insect species (Ward and Johnson, 2013) and seems to be less specialized than *G. physocarpus*. The floral nectaries of both species are formed by five corona lobes, which are partly covered in *G. fruticosus* but not in *G. physocarpus* (Burger et al., 2017). The pollen is packaged in pollinia and clamped to the tarsi of the pollinating insects.

For this study, *G. physocarpus* and *G. fruticosus* plants were grown from seed in the botanical garden of the University of Ulm, Germany. Approximately 20 individual plants of each species were available every year. *Vespula germanica* (Fabricius 1793) and *V. vulgaris* (Linnaeus 1758) wasps and *Apis mellifera* Linnaeus 1758 honeybees were used for electrophysiology and feeding experiments. The wasps were caught when leaving or entering nests located at the campus of the University of Ulm. Honeybees were taken from three hives hosted by local beekeepers of the Bezirks-Imkerverein Ulm e.V.

### Nectar cardenolides analysis

#### Sample preparation

For each nectar sample, between 5 and 200 µl nectar was collected using glass capillaries with a loading capacity of 2 or 5 µl (minicaps, ISO 7550, DURAN, Hirschmann, Germany) and stored in Eppendorf tubes (Eppendorf, Germany) at −40°C. Inflorescences were cut and stored in plastic bags at −40°C. The plant material was then freeze dried and stored at −20°C until further sample preparation. Ten nectar samples from *G. physocarpus* and six from *G. fruticosus* as well as four flower samples from each plant species were prepared for compound identification and quantitative comparison between samples.

The nectar was extracted in 80% methanol in a 1:4 ratio and placed into 0.3 ml LC-MS vials (Thermo Fisher Scientific, Singen, Germany) for chemical analysis. For flower extracts, two flowers were put into a vial, the mass of the flowers was determined, and then they were extracted in 1 ml 80% methanol overnight. The following day, the samples were put into a Fisherbrand R sonicator (Thermo Fisher Scientific, USA) for 10 min. Afterwards, the fluid was transferred into Eppendorf tubes and centrifuged for 1 min. The supernatant was transferred into 2 ml LC-MS vials.

#### Compound identification and quantitative comparison

To compare cardenolides in the nectar and flowers of *G. physocarpus* and *G. fruticosus*, extracts were analysed using liquid chromatography (LC) coupled with electrospray ionisation mass spectroscopy (ESI-MS). The analyses were performed at the Royal Botanic Gardens, Kew, UK. A Thermo Scientific Dionex UltiMate 3000 (Thermo Fisher Scientific) was coupled to a Thermo Scientific Velos Pro mass spectrometer (Thermo Fisher Scientific). The column used for separation of the compounds was a Phenomenex Luna 3 µm C18(2) 100 Å (150×3.0 mm, Macclesfield, UK) using a 400 µl min^−1^ mobile phase of 0% A:90% B:10% C (*t*=0) to 90% A:0% B:10% C (*t*=20 to 25 min) returning to 0% A:90% B:10% C (*t*=27 to 30 min), where A is methanol, B is water and C is acetonitrile+1% formic acid. One run was 30 min at a constant temperature of 30°C. The injection volume was 5 µl.

To facilitate compound identification, high resolution ESI-MS data were recorded on one sample for each species using an Orbitrap Fusion^TM^ mass spectrometer (Thermo Fisher Scientific) coupled to a Thermo Accela LC system (Thermo Fisher Scientific) conducting chromatographic separation of 5 µl injections on the same column as described above. The Orbitrap used the same mobile phase gradient, column temperature and flow rate as described for the LC-MS. For tentative identification of compounds, the molecular formula empirically determined from pseudomolecular ion with *m*/*z* [M+H]^+^ was compared with that of [M+H]^+^ molecular ions for known cardenolides of *Gomphocarpus* recorded in the Combined Chemical Dictionary database (http://ccd.chemnetbase.com/faces/chemical/ChemicalSearch.xhtml) (Table 1).

**Table 1.**
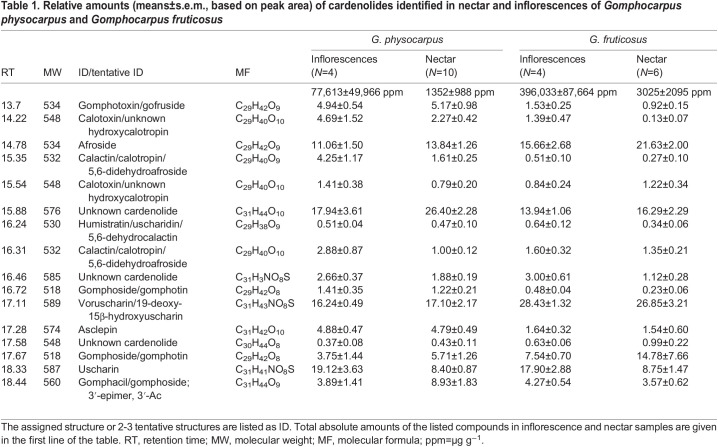
Relative amounts (means±s.e.m., based on peak area) of cardenolides identified in nectar and inflorescences of *Gomphocarpus physocarpus* and *Gomphocarpus fruticosus*

Absolute amounts were calculated using afroside as a standard. Afroside (1.3 mg) was isolated from the cardenolide fractions of *Gomphocarpus* corolla samples (see section Isolation of cardenolide fractions below; methods following Green et al., 2011) and the structure was confirmed by comparison of nuclear magnetic resonance (NMR) data with literature (Cheung et al., 1981). For the calculation of total absolute amounts, the total peak area per sample was corrected for the sample dilution (see section Sample preparation above) and converted to ppm (=μg g^−1^) based on a calibration curve. The dilution factor for flower extracts was determined based on the mass of the flower sample and extraction solvent.

We analysed semi-quantitative differences in the composition using Primer 6.1.15 (Clarke and Gorley, 2006). We calculated the relative amounts of single components with respect to the total amount of peak areas in a sample and square-root transformed the data. Based on pairwise Bray–Curtis similarities, we visualized the similarities and dissimilarities among the samples using non-metric multidimensional scaling and performed an ANOSIM (9999 permutations) to test for differences between species and between floral types (nectar and inflorescences) using a two-way crossed design. We evaluated the contribution of single substances to the observed dissimilarities between species and floral types using a SIMPER analysis.

#### Isolation of cardenolide fractions

For the isolation of cardenolides, 15 g of freeze-dried, milled *G. physocarpus* flowers and 25 g of freeze-dried, milled *G. fruticosus* flowers were extracted in 450 or 750 ml methanol, respectively, for 24 h under occasional stirring and then filtered through Whatman Grade 1 filter paper. Extracts were dried on a rotary evaporator until complete removal of the solvent.

A cardenolide and a flavonoid fraction were prepared from the extracts of both *Gomphocarpus* species using flash chromatography on a Biotage Isolera One (Biotage, Sweden) system with a SNAP Ultra C18 cartridge. Extracts were re-dissolved in 5 ml of 80% methanol, and loaded on top of the column. The solvent gradient run comprised two column volumes of 10% methanol in water followed by a linear gradient from 10% methanol in water to 100% methanol over nine column volumes and a final two column volumes of 100% methanol at a constant flow rate of 50 ml min^−1^. UV absorbance spectra of the eluate were monitored at 220 nm (peak absorbance of *Gomphocarpus* cardenolides) and 350 nm (flavonoid peak absorbance), and the eluate was fractionated by distinct UV absorbance peaks. Fractions were analysed via HPLC-MS (see conditions described for analysis on a Velos-Pro mass spectrometer, in section ‘Compound identification and quantitative comparison’ above), and fractions containing cardenolides or flavonoids combined separately.

### Feeding experiments

In the feeding experiments, bees and wasps were offered feeding solutions for 5 days to test for avoidance/preference behaviour at different time points and to measure the survival rate. We performed different feeding experiments (bees *N*=13–18 per group; wasps *N*=14–15 per group), which involved testing responses to *G. physocarpus* nectar, isolated cardenolides in different concentrations, and a cardenolide-free fraction (flavonoid fraction). Not all treatments were tested with both organisms and the experiments focused on the feeding behaviour of honeybees. The test chambers with individual bees or wasps were kept at 26°C and in darkness to avoid side preferences towards light sources. Each test chamber contained moistened paper tissue.

Approximately 50–70 µl of a feeding solution was offered in small vials (0.5 ml tubes, Eppendorf) that were prepared with three holes. One hole was positioned close to the tip for drinking, the second was for pressure equalization and the third (in the lid) was used to refill the tubes. The insects were fed with sugar water offered in feeding vials before an experiment started. Vials filled with sugar water and prepared in the same way as the other feeding vials were used to check the evaporation rate.

#### Nectar bioassays

Bees were given the choice between (1) *G. physocarpus* nectar and sugar water (*N*=25) and (2) only sugar water (*N*=17). The sugar composition (composed of 92% sucrose, 3% glucose and 5% fructose; Burger et al., 2017) and the sugar concentration (20 to 30%, depending on the used sample) of the sugar water resembled the nectar samples. Pooled nectar samples (1–2 ml) were used. The bees were kept separately in *Drosophila* tubes (height 10 cm, diameter 4.5 cm) closed with corresponding sponges (height 3 cm). Two feeding vials (described above) and two Teflon tubes (length 4 cm, inner diameter 3 mm) to allow airflow were inserted into the *Drosophila* tube and fixed with the sponge. To avoid site preferences through attraction towards indirect light sources, the test chambers were placed into a plastic box that were covered with aluminium foil. A pump with a flow rate of 250 ml min^−1^ was connected to refresh the air in the box.

#### Cardenolide bioassays

No-choice feeding experiments with isolated cardenolides and a cardenolide-free fraction (flavonoid fraction) were performed with bees and wasps. The isolated fractions were dissolved in distilled water while heating (45°C) and sonicating the sample alternately. Then, the solution was further diluted with sugar water to obtain a final solution with a sugar concentration of 30% (sugar composition see above). The metabolite concentrations were adjusted to facilitate testing high, medium and low ecologically relevant concentrations. The concentrations corresponded to the maximum, medium (mean of maximum and minimum) and minimum concentration found in the analysed nectar samples based on peak areas: *G. physocarpus* low: 25.79 ppm, medium: 2656.87 ppm, high: 9212.01 ppm; *G. fruticosus* low: 319.28 ppm, high: 11,225.61 ppm. Not all solutions were tested with both bees and wasps and the experiments focused on the feeding behaviour of honeybees. The insects were kept in small wooden boxes that allowed the insects to move more freely and for easier handling of the feeding solutions as compared with the test chambers used for nectar bioassays.

#### Analysis of feeding experiments

The feeding tubes were weighed every day using a high precision weighing scale (accuracy minimum 1 mg) to determine the consumed amount of feeding solutions. The feeding tubes were refilled if necessary. To calculate the total consumed, the values were summed until the individual bee or wasp died. To correct for evaporation loss, the values were corrected for the mean loss of control tubes that were filled with sugar water and kept in the same conditions. The mass of the consumed nectar and sugar water was compared using a *t*-test in SPSS 26.

Analysis of survival was performed using the Kaplan–Meier method implemented in R (https://www.r-project.org/) with the packages survival (https://CRAN.R-project.org/package=survival) and survminer (https://CRAN.R-project.org/package=survminer). Pairwise comparisons among survival with correction for multiple tests (log-rank test) were performed with the function pairwise_survdiff (https://CRAN.R-project.org/package=survival) with *P*-values adjusted for multiple comparisons (Bonferroni–Holm method).

### Floral volatiles analysis

#### Chemical composition

Scent was collected using dynamic headspace methods. Fifteen inflorescences were cut off from the plant and put into an oven bag (Toppits, Germany) for each sample. Adsorbent filters were filled with 1.5 mg Carbosieve^TM^ (60/80 mesh, Sigma-Aldrich) and a mixture of 1.5 mg Carbotrap^TM^ (20/40 mesh, Sigma-Aldrich) and 1.5 mg Tenax^TM^ (60/80 mesh, SUPLECO), separated with a layer of glass wool. Carbotrap^TM^ and Tenax^TM^ have a high affinity for lipophilic to medium-polar compounds and medium-molecular weight organic compounds, whereas Carbosieve^TM^ traps has higher affinity for low-molecular weight organic (C2–C5 n-alkanes) and polar compounds. The adsorbent filter was connected to a pump through a silicon tube. The part of the filter filled with Tenax and Carbotrap was directed to the bag with flowers to function as a pre-filter for Carbosieve. The bag was enriched with scent for 30 min and the contents then sucked out for 1 h with a flow of 100 ml min^−1^. An empty bag was used as a blank control. Green parts from 15 inflorescences were used as vegetative controls. In addition, headspace samples from nectar were taken for chemical analyses. The nectar was collected from several flowers and inflorescences of one individual with a total amount of 40 µl per sample. The nectar was placed on filter paper and the scent was collected using the same method as described above. Blank controls were collected from filter paper wetted with 40 µl water. All samples were stored at −20°C.

The headspace samples were analysed using gas chromatography coupled to mass spectrometry (GC-MS). In total, 17 samples of *G. physocarpus* inflorescences, 3 samples of *G. physocarpus* nectar, 26 samples of *G. fruticosus* inflorescences, 3 samples of *G. fruticosus* nectar, 5–7 vegetative control samples of each species, 15 blank samples for the inflorescences and 2 blank samples for the nectar were analysed. A gas chromatograph (Agilent Technologies 7890B) equipped with a polar column (DB-Wax, 30 m long, 0.25 mm inner diameter, 0.25 µm film thickness, Agilent) coupled to a mass spectrometer (Agilent Technologies 5977A) was used. The GC-MS was equipped with a ChromatoProbe Kit and a thermodesorption unit (TDU, Gerstel, Germany). The starting temperature of the oven was 40°C, held for 2 min, and then raised at 6°C min^−1^ to a final temperature of 240°C. The mass spectra were recorded with 70 eV of *m*/*z* 30–350. A cooled injection system was used to cryofocus the analytes. It was cooled down with liquid nitrogen to −100°C.

We confirmed the identification of individual components by comparison of both the mass spectrum and GC retention data with authentic standards. Active compounds were assigned to GC-MS runs by comparing the elution sequence and retention indices. Amounts of the compounds were calculated using AMDIS 2.71 (Automated Mass Spectral Deconvolution and Identification System). To estimate the absolute amount of the compounds in headspace samples, we injected 0.1 µg of a standard (dodecane 100 µg ml^−1^ hexane) as an external standard.

To identify flower-specific volatiles, the background (compounds recorded in blank controls) was subtracted from the plant samples. Subsequently, compounds identified as contaminants were excluded from further analyses. The inflorescence samples were also compared with the vegetative samples and only volatiles that occurred in higher amounts or only in inflorescence samples were classified as flower constituents.

We analysed the semi-quantitative differences in the scent bouquets using Primer 6.1.15 (Clarke and Gorley, 2006). We calculated the relative amounts of single components with respect to the total amount in a sample and square-root transformed the data. Based on pairwise Bray–Curtis similarities, we visualized the similarities and dissimilarities among the samples using non-metric multidimensional scaling and performed an ANOSIM (9999 permutations) to test for differences between species and between floral types (nectar and inflorescences) using a two-way crossed design. We evaluated the contribution of single substances to the observed dissimilarities between species and floral types using a SIMPER analysis.

#### Electrophysiology experiments

To identify the compounds that were detectable by flower visitors, we performed gas chromatography coupled with electroantennography (GC-EAD). The GC-EADs were conducted with antennae of *V. germanica* wasps and *A. mellifera* bees exposed to the floral odour of *G. physocarpus* and *G. fruticosus*. Not all treatments were tested with both organisms and the experiments focused on electrophysiological responses of wasps. In total, 8 analyses with *G. physocarpus* and wasp antennae were performed, 7 with *G. physocarpus* and bee antennae and 6 with *G. fruticosus* and wasp antennae.

The GC-EAD system consisted of a gas chromatograph (Agilent Technologies 7820A) equipped with a flame ionisation detector (FID) and an electroantennogram detector (EAD). A thermal desorption injector was connected to the system. The chromatoprobe samples were injected in splitless mode into the GC at an initial temperature of 40°C (injector temperature 200°C). The oven temperature was held for 1 min and then raised at 10°C min^−1^ to a final temperature of 240°C, which was held for 5 min. We used a polar column (DB-Wax, 30 m long, 0.25 mm inner diameter, 0.25 µm film thickness, Agilent) and the carrier gas was hydrogen (2 ml min^−1^). The GC effluent was split using a column split to a prepared antenna and the FID separately. The antennae were cut off at the tip and base and fixed between two glass capillaries filled with insect Ringer solution (8.0 g l^−1^ NaCl, 0.4 g l^−1^ KCl, 0.4 g l^−1^ CaCl_2_) connected to gold electrodes, closing an electric circuit. Before the antenna was cut off, individuals were cooled on ice for several minutes until they stopped moving. For simultaneous responses of FID and EAD, the GC effluent was split (split ratio 1:1). The signals were recorded with the GCEad-1.2.5 program (Syntech, Buchenbach, Germany). If a substance was active in at least three runs it was considered as electrophysiologically active.

#### Behavioural responses to scent compounds

The attractiveness of electrophysiologically active substances of *G. physocarpus* were tested in a behavioural choice experiment with *V. germanica* wasps. The synthetic mixture (Table S1) consisted of compounds identified as electrophysiologically active. The amounts were adjusted until they resembled the natural samples in quantity and quality (based on preliminary samples). To do so, 50 µl of the synthetic mixture was applied on a filter paper and headspace samples were collected using the same methods as described above. Diethyl phthalate was used as a solvent because of its low molecular weight (near-odourless) and its use as a solvent for polar compounds (Nevo et al., 2015).

The bioassay was performed with freely flying *V. germanica* wasps in a flight tent (Aerarium, 60×60×90 cm, Bioform, Germany). The flight tent was positioned over an entrance hole of a wasp nest according to Lukas et al. (2020). The nest was located in the botanical garden of the University of Ulm. Approximately 10 wasps were inside the tent at any time. After an acclimatization phase of 1 h, the synthetic mixture and diethyl phthalate (99% purity, Sigma-Aldrich) as a solvent control were offered to the wasps. A filter paper was impregnated with 50 µl of the fluid at the beginning and after 30 min and put into a closed oven bag (Toppits). Small holes were cut all over the bag, which was connected to a pump (air flow 900 ml min^−1^) to allow an airstream. Landings and approaches of the wasps were recorded for 1 h. The position of the test solutions was changed after 30 min. The choice of the wasps was compared using an exact binomial test in SPSS 26.

## RESULTS

### Nectar cardenolides

#### Chemical composition

The chemical composition of nectar and inflorescences was similar for *G. physocarpus* and *G. fruticosus* but compounds differed quantitatively between the two species (Table 1). All compounds occurred in both the inflorescences and the nectar of both species. In total, we detected 19 compounds, of which 16 were assigned to be cardenolides. The structures of three cardenolides (afroside, asclepin and uscharin) were determined, 10 were assigned to two or three tentative structures based their molecular formulae and comparison with data in the Combined Chemical Dictionary (http://ccd.chemnetbase.com/faces/chemical/ChemicalSearch.xhtml) on known structures from this plant group, and three were not identified (unknown cardenolides). All compounds are listed in Table 1, and the first tentative ID is used in the text.

**Fig. 2.**
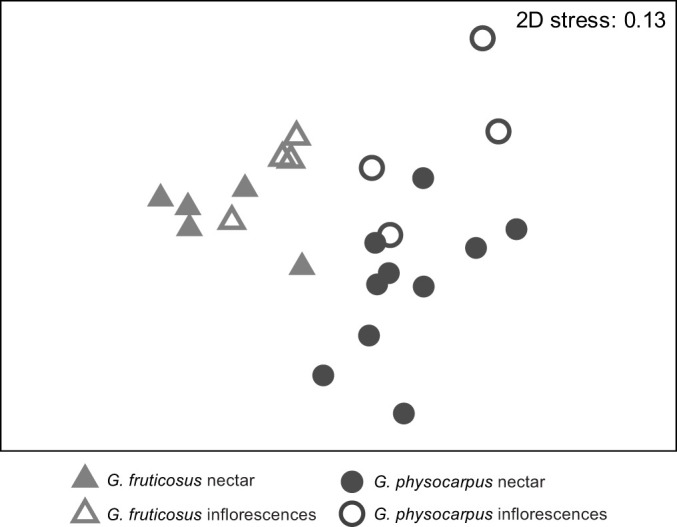
**Comparison of the composition of cardenolides in the nectar (filled symbols) and inflorescences (open symbols) of *Gomphocarpus*** ***physocarpus*** **(dark grey) and *Gomphocarpus fruticosus* (light grey)**. Multi-dimensional scaling plot based on the Bray–Curtis index (ANOSIM, species: *R*=0.70, *P*<0.001, floral type: *R*=0.30, *P*<0.05, *n*=23).

The two milkweed species differed in the overall relative amounts of nectar cardenolides (ANOSIM, species: *R*=0.70, *P*<0.001, *n*=23; Fig. 2). Across species, the semi-quantitative composition of nectar and inflorescences samples were almost the same (ANOSIM, floral type: *R*=0.30, *P*<0.05, *n*=23; Fig. 2). Voruscharin mostly characterized the inflorescences of both species and the nectar of *G. fruticosus* (SIMPER analysis). The nectar of *G. physocarpus* were mostly characterized by an unknown cardenolide (RT 15.88).

#### Nectar consumption

**Fig. 3.**
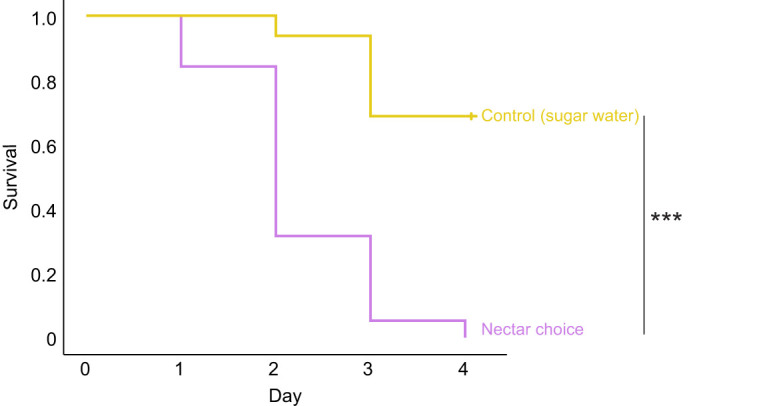
**Cumulative survival of *Apis mellifera* honeybees in nectar choice feeding experiments.** Bees of the nectar treatment group (*n*=25) were offered the choice between *G. physocarpus* nectar and sugar water, the control group (*n*=17) was given sugar water only (log rank, ***P<0.001).

**Fig. 4.**
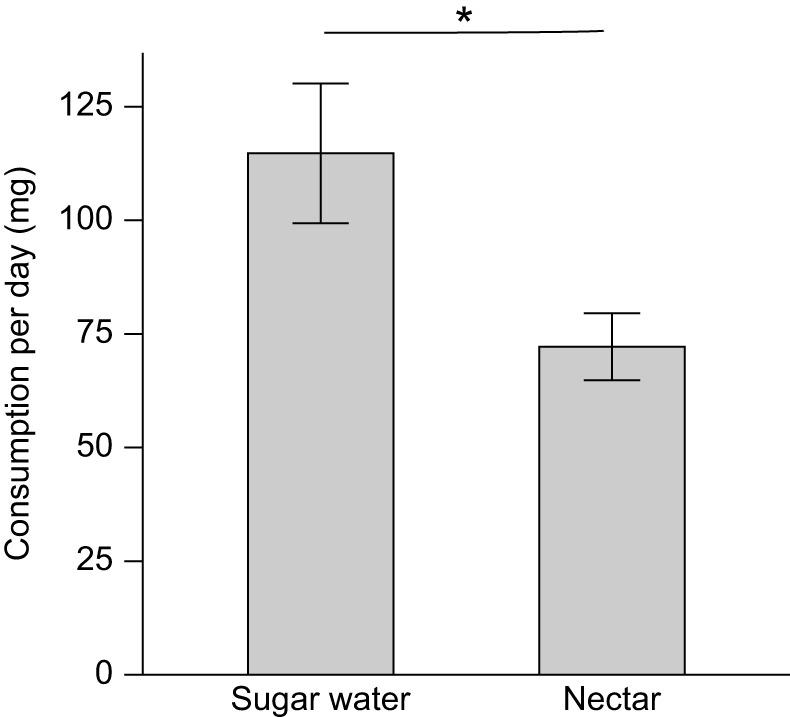
**Mean daily consumption of *Gomphocarpus physocarpus* nectar and sugar water in feeding choice experiments with honeybees.** Data shown are means±s.e.m. (*t*-test: *n*=25 bees, *P<0.05).

The group of bees that had a choice between *G. physocarpus* nectar and sugar water died significantly earlier than the control group, which was fed with sugar water only (log rank: χ^2^=26.4, *P*<0.001, *n*=17–25 per group) (Fig. 3). Although the bees in the nectar treatment group consumed significantly more sugar water than nectar per day (*t*-test: *n*=25 bees, *t*=2.50, *P*<0.05), they did not stop drinking nectar entirely (Fig. 4).

#### Cardenolide consumption

**Fig. 5.**
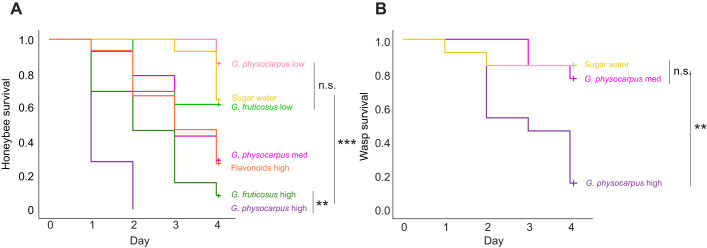
**Cumulative survival of *Apis mellifera* honeybees and *Vespula*** ***germanica*** **wasps in cardenolide feeding experiments.** (A) Honeybees and (B) *V. germanica* wasps were fed with isolated fractions of cardenolides of *G. physocarpus* (bees: high *n*=18, medium *n*=14, low *n*=14; wasps: high *n*=14, medium *n*=15) or *G. fruticosus* (bees: high *n*=13, low *n*=13) in different natural concentrations, or flavonoids of *G. physocarpus* (bees: *n*=15), all dissolved in sugar water. The control group (bees: *n*=14, wasps: *n*=14) were fed with sugar water (log rank pairwise comparisons, n.s.: *P*>0.05, **P<0.01, ***P<0.001, see also Table S2).

In the no-choice experiments involving feeding on solutions containing cardenolides, the survival rates of honeybees differed significantly between the different treatment groups (log rank: χ^2^=83.9, *P*<0.001, *n*=13–18 per group) (Fig. 5A; Table S2). All bees that were fed with the high cardenolide concentration of *G. physocarpus* died after 1 or 2 days (50% of the bees died after 1 day; total consumption until death: mean±s.e.m. 83.70±6.53 mg of 9212.01 ppm cardenolides). The bees fed with medium concentrations survived significantly longer but still showed a high mortality (3 days, 218.77±21.00 mg of 2656.87 ppm cardenolides). The bees fed with cardenolides isolated from *G. fruticosus* survived significantly longer but many also died within the duration of the experiment (2 days, mean consumption of 113.24±11.49 mg of 11,225.61 ppm cardenolides). Honeybees fed with low cardenolide concentrations of both *Gomphocarpus* species showed a low mortality.

Wasps also suffered mortality when they consumed high cardenolide concentrations (4 days, 159.50±19.26 mg of 9212.01 ppm cardenolides), but there was no significant difference between the medium concentration and sugar water treatment groups (log rank: χ^2^=17, *P*<0.001, *n*=14–15 per group) (Fig. 5B).

### Floral volatiles

#### Chemical composition

**Fig. 6.**
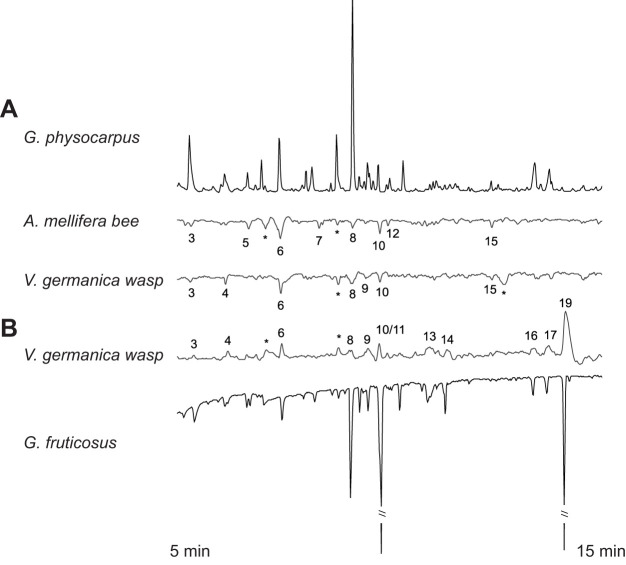
**Representative GC-EADs of headspace volatiles of *Gomphocarpus* spp. tested with *Apis mellifera* and *Vespula germanica*.** (A) *Gomphocarpus physocarpus* and (B) *G. fruticosus* inflorescences were tested with antennae of (A) *A. mellifera* honeybees and (A,B) *V. germanica* wasps (shown sensitivity FID 50 mV, EAD 1 mV). Electrophysiological responses to floral compounds are numbered (*responses to compounds found in blank controls; numbers correspond to numbers given in Table 2).

**Fig. 7.**
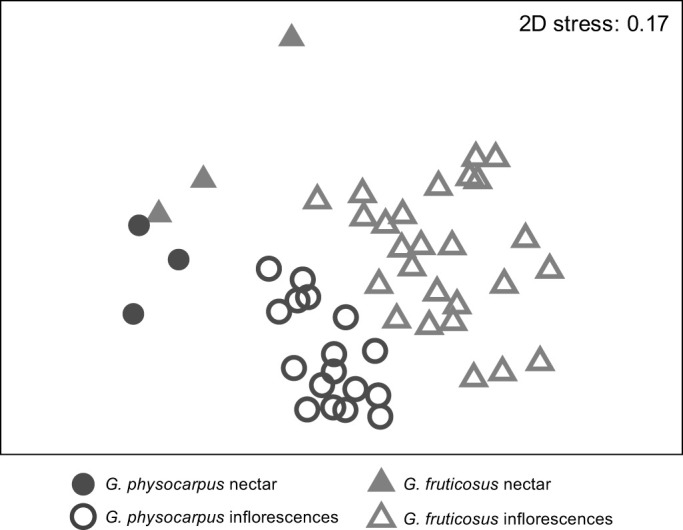
**Comparison of volatile compounds emitted by inflorescences (open symbols) and nectar (filled symbols) of *Gomphocarpus*** ***physocarpus*** **(dark grey) and *Gomphocarpus fruticosus* (light grey).** Multi-dimensional scaling plot based on the Bray–Curtis index (ANOSIM, species: *R*=0.58, *P*<0.001, floral type: *R*=0.91, *P*<0.001, *n*=49).

The GC-EAD experiments of *G. physocarpus* and *G. fruticosus* tested with antennae of *V. germanica* and *A. mellifera* revealed 21 inflorescence volatile compounds that were electrophysiologically active (Table 2, Fig. 6). The two milkweed species differed in the overall relative amounts of electrophysiological active floral scent compounds (ANOSIM species, *R*=0.58, *P*<0.001, *n*=49; Fig. 7). Nectar and inflorescence samples differed significantly across species (ANOSIM floral type, *R*=0.91, *P*<0.001, *n*=49; Fig. 7). Acetic acid mostly characterized the inflorescences and nectar of *G. physocarpus*. In *G. fruticosus*, the inflorescences were mainly characterized by benzyl nitrile and the nectar by phenol (SIMPER analysis).

**Table 2.**
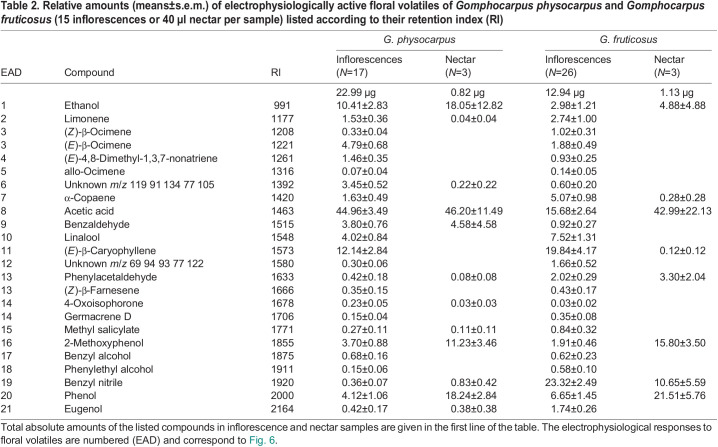
Relative amounts (means±s.e.m.) of electrophysiologically active floral volatiles of *Gomphocarpus physocarpus* and *Gomphocarpus fruticosus* (15 inflorescences or 40 µl nectar per sample) listed according to their retention index (RI)

#### Behavioural responses to floral volatiles

**Fig. 8.**
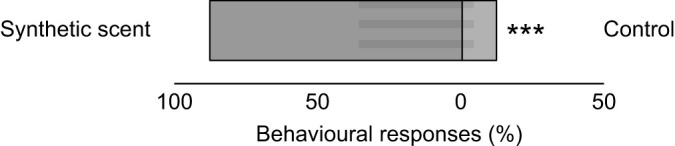
Behavioural responses of *Vespula germanica* wasps to a synthetic mixture resembling the scent of *Gomphocarpus physocarpus* inflorescences tested in a choice against a solvent control (exact binomial test: **P<0.01, *P<0.05). The shaded area illustrates the relative proportion of landings versus approaches only.

In the choice experiments testing the attractiveness of the synthetic scent mixture resembling *G. physocarpus* floral scent against a solvent control, 22 out of 25 *V. vulgaris* wasps were attracted by the synthetic mixture (exact binomial test: *P*<0.001) (Fig. 8). Of these, 13 individuals approached and nine landed on the synthetic scent, and two approached and one landed on the control.

## DISCUSSION

Our experiments show that chemical traits of *G. physocarpus* flowers moderate this specialized pollination system. Wasp-pollinated *G. physocarpus* and bee-pollinated *G. fruticosus* contained similar cardenolides in the nectar but they differed in relative proportions of the individual compounds. *Vespula germanica* wasps were not affected by relatively high amounts of cardenolides, which, in contrast, caused reduced feeding and high mortality in *A. mellifera* honeybees. In addition, floral volatiles play a key functional role in attracting *V. germanica* wasps to flowers of *G. physocarpus*.

### Nectar cardenolides filter non-pollinating honeybees

We performed feeding experiments with natural nectar samples and with an isolated cardenolide fraction to account for interaction effects between different secondary compounds. Previous experiments with individual compounds have shown different toxic effects for different compounds and concentrations, making it difficult to predict pollinator response to natural compositions of nectar compounds. Although it is challenging to collect sufficient nectar and plant material for behavioural experiments, it is important to test naturally occurring compounds to understand the role of nectar as a filter mechanism.

The feeding experiments showed that the nectar of the wasp-pollinated *G. physocarpus* was highly toxic to honeybees. Although the bees of the treatment group did not solely feed on nectar (sugar water was additionally offered), they died significantly earlier than the control group, which was fed with sugar water only. In addition, bees drank significantly more sugar water than nectar during the experiment, implying that the nectar was distasteful for them. It is also possible that they learned to avoid it because of its effects on their physiology, but we did not observe increasing rejection over time in our experiments (H.B., unpublished results). Some secondary metabolites found in nectar are deterrent or toxic only at concentrations above their natural occurrence in nectar, e.g. caffeine (Wright et al., 2013), but in *Gomphocarpus* spp. they are biologically active at natural concentrations. However, the bees did not stop consuming the toxic nectar entirely, although they had the choice to consume sugar water with the same sugar concentration. Generalist bee species often have a poor acuity for the detection of nectar toxins (Tiedeken et al., 2014), which could explain the observed feeding behaviour. In addition, nectar contains further nutritional compounds such as amino acids besides sugars (Baker and Baker, 1986; Power et al., 2018), and the nectar provided may have been perceived as a higher valuable food source compared with the control, which provided only carbohydrates.

The cardenolide feeding experiments showed that the toxicity was concentration dependent for *A. mellifera* honeybees and *V. germanica* wasps. Honeybees showed high mortality in response to cardenolide concentrations (e.g. medium concentration) that did not lead to mortality in wasps. A defensive filtering function of nectar has also been revealed in other specialized plant–pollinator interactions. *Pachycarpus grandifloras*, a milkweed pollinated almost exclusively by *Hemipepsis* spider-hunting wasps, has bitter tasting nectar that is unpalatable for bees but not for the pollinating wasps (Shuttleworth and Johnson, 2009b). In another example, short-tongued bees that attempt to rob *Aconitum* flowers encounter highly deterrent diterpene alkaloids in the nectar, whereas the pollinating long-tongued species can tolerate higher concentrations of these alkaloids (Barlow et al., 2017). Tiedeken et al. (2016) also reported the selective toxicity of diterpenoids in the nectar of *Rhdodendron ponticum*. Honeybees exposed to the compounds at natural concentrations died within hours, whereas bumblebees, the preferred pollinators, were unharmed after 30 days. Our data suggest a similar role for nectar cardenolides of *G. physocarpus*, and the adaptation of wasps to tolerate relatively high amounts of these toxins appears to mediate the specialization of *G. physocarpus* for wasp pollination.

As *V. germanica* wasps occur only in the non-native range of *G. physocarpus*, ideally the hypothesis needs to be tested with native southern African wasp species such as *Polistes fastidiosus* or *Belanogaster dubia* (Coombs et al., 2009), although this would be challenging because experimental protocols for these species are not well established. However, the pollinator species in the non-native ranges effectively compensate for native pollinators. Although *G. physocarpus* is specialized for wasp pollination (functional group of medium-sized vespid wasps), the pollination system appears to be generalized across different species of Vespidae. In contrast, other milkweed species of South Africa are typically pollinated by only one or two pompilid wasp species (Shuttleworth and Johnson, 2012). Compared with these highly specialized systems and even compared with many other plant species (Ollerton et al., 2003), the pollination success and fruit set in *G. physocarpus* is comparatively high, which may reflect the broad spectrum of insects that can function as its pollinators. The wasp-pollination system generalized at the species level and the presence of functionally similar wasps in other parts of the world are assumed to facilitate the capacity for *G. physocarpus* to colonize new areas and expand its distribution range. Recent studies also suggest a degree of plasticity in the expression of nectar toxins in non-native ranges used to filter pollinators depending on the priority, such as when challenged by increased herbivory or when pollination services have changed or are limited (Egan et al., 2016, 2022).

*Vespula germanica* wasps showed ill effects only after consuming solutions with the highest natural cardenolide concentrations. That wasps show any ill effects from cardenolides seems paradoxical given that they are the main pollinators, although honeybees showed similar responses to high concentrations of nectar toxins from *G. fruticosus*. However, it may be critical for plants to deter less-effective pollinators with cardenolides, even if there are mild effects on the main pollinators – a mechanism also reported in *Aconitum* spp. (Barlow et al., 2017). Toxic effects due to high cardenolide concentrations are observed even for monarchs that are highly specialized and depend on milkweeds for their development (Zalucki et al., 2001). Therefore, females preferentially oviposit on milkweed plants with intermediate levels of cardenolides (Oyeyele and Zalucki, 1990; van Hook and Zalucki, 1991). *Danaus plexippus*, for example, oviposited mostly on *G. fruticosus* plants that were, on average, 20% lower in foliar cardenolides than the overall population average (Oyeyele and Zalucki, 1990). Similarly, a dominant floral visitor of *Asclepia* plants, *B. griseocollis* bees, avoided high levels of cardenolides in a behavioural experiment, which suggests that they have an innate ability to avoid the most toxic milkweeds (Villalona et al., 2020). In congruence, honeybees and wasps might also select plants with lower concentrations of cardenolides for nectar foraging on the studied milkweed species. Levels of cardenolides in milkweed can vary substantially among plants, e.g. with stressed plants producing more cardenolides (Rasmann et al., 2009). We also found a wide range of natural concentrations in the nectar of both species. This finding might explain observations in the field that honeybees occasionally visit *G. physocarpus* flowers. That the plants are attractive under certain circumstances might depend on the cardenolide concentration. Honeybees are able to remove pollinia from *G. physocarpus* flowers, but it is unclear whether they are able to reinsert them for pollination (Forster, 1994). Filter mechanisms against honeybees and other visitors allow specializations to functional pollinator groups, in this case to wasps, which can increase the pollination efficiency of plants through effective pollen transfer (Fenster et al., 2004).

It was also unexpected that honeybees were negatively affected by nectar from *G. fruticosus* cardenolides, given that they pollinate this species. However, this is known in other pollinator–plant interactions. For example, Barlow et al. (2017) report avoidance of some flowers of *Aconitum* spp. by the pollinator *B. hortorum* attributable to diterpenoid alkaloids known to be toxic to *Bombus* spp., but this effect is dependent on the concentration of the toxin in the nectar, which varies among flowers of the same and neighbouring plants. In the present study, cardenolides of *G. physocarpus* were more toxic for honeybees than those of *G. fruticosus*. If pollinators avoid high levels of nectar toxins, plants would not necessarily suffer from reduced pollination if it conferred any fitness benefit to the plant. Toxins in nectar may also reduce damage from antagonistic herbivores or colonization by microorganisms (Martin et al., 2022). Generalist visitors such as honeybees encounter these toxins often, given the large numbers of plants that have toxins in their nectar (Stevenson, 2020). However, altering food sources can dilute the effects (toxin dilution), and dietary mixing of favourable and unfavourable food sources seems to be a common strategy in insects to complement nutrient deficiencies or to mitigate harmful secondary metabolites. For example, diet mixing is shown for pollen collection behaviour of bees (Eckhardt et al., 2014) or nectar intake by herbivorous insects (Singer et al., 2002). We also cannot exclude that honeybees in the non-native range of *G. fruticosus* are less sensitive to the cardenolides found in *G. fruticosus* nectar, but it is likely that the successful invasion outside the native range is attributable, at least in part, to their generalised pollinator requirement (Ward and Johnson, 2013). The visitor spectrum of *G. fruticosus* is dominated by bees in the native range (Burger et al., 2017), but different wasp species were observed to visit *G. fruticosus* in non-native Australia (Ward and Johnson, 2013). Although the site of nectar accumulation is partly covered in *G. fruticosus* flowers functioning as morphological filter against short-tongued insects such as wasps, wasps can nevertheless reach standing crops of nectar. If bee pollinators are rare in a region or deterred by high cardenolide concentrations, wasps might compensate for fewer visits of bee pollinators.

The chemical analysis showed significant differences between the composition of cardenolides of the two plant species *G. physocarpus* and *G. fruticosus*. Species-specific compounds are typical for nectar and pollen (Palmer–Young et al., 2019); however, in this system, both milkweed species had similar nectar components, but they differed in their relative and absolute amounts. *Gomphocarpus fruticosus* had higher absolute amounts of cardenolides compared with *G. physocarpus*, but was less toxic in feeding experiments. Consequently, the different levels of toxicity were likely caused by different concentrations of individual compounds that differed in their toxic effects on invertebrates. Different cardenolides can have different toxic effects. For example, convallatoxin is highly deterrent to bees, whereas ouabain shows only a tendency to cause effects in higher concentrations and digitoxin showed no effect at all (Detzel and Wink, 1993). Similar variation in bioactivity occurs in other systems. For example, and as referred to above, in *R. ponticum* nectar, grayanotoxin 1 causes honeybee mortality at naturally occurring concentrations, whereas grayanotoxin 3, which differs from the former compound by just one hydroxyl substitution, is not toxic at the same concentration as grayanotoxin 1 (Tiedeken et al., 2016).

Previously tested cardenolides were studied in regard to *Asclepias* systems, although they do not naturally occur in *Asclepias* plants but are the only commercially available cardenolides. Although the toxicity of individual compounds remains unclear, it is also an open question how frequently floral visitors are faced with highest cardenolide amounts as the toxicity also depends on the consumed amount of nectar. *Gomphocarpus physocarpus* produces larger nectar volumes with slightly higher sugar concentrations in comparison to *G. fruticosus* (Ward and Johnson, 2013). A comprehensive field study comparing cardenolide amounts between individuals of both species is needed to better describe the natural situation.

Nectars often have a distinct chemical composition compared with other plant tissue (Manson et al., 2012; Palmer-Young et al., 2019), whereby cardenolides are normally present in high concentrations in the leaves of the plants as they act as a defence against herbivores (Agrawal et al., 2012). In *G. physocarpus* and *G. fruticosus*, the inflorescences and the nectar differed only slightly in the composition of cardenolide compounds. We found smaller absolute cardenolide amounts in the nectar compared with other plant species (Manson et al., 2012), but they were highly toxic according to the feeding experiments. The high toxicity is a hint that the cardenolides in the nectar of *G. physocarpus* and *G. fruticosus* plants are not only a by-product of leaf defence but also have adaptive functions in the nectar.

### Floral volatiles attract pollinating wasps

Differences in the floral scent bouquets between plant species allow floral visitors to discriminate between different host and non-host plants. Floral scent is also an important attractant in the majority of wasp-pollinated plants (Brodmann et al., 2008, 2009; Shuttleworth and Johnson, 2009b). Our chemical analyses showed that the semi-quantitative composition of electrophysiologically active compounds differed between *G. physocarpus* and *G. fruticosus*. A previous study demonstrated that the scent of *G. physocarpus* was significantly more attractive for wasps than that of *G. fruticosus* (Burger et al., 2017). The scent differences enable the floral visitors to discriminate between both species based on olfactory cues (Burger et al., 2017). The scent of *G. physocarpus* was mainly characterized by acetic acid and that of *G. fruticosus* by benzyl nitrile. Benzyl nitrile is a typical component of floral scents (Knudsen and Gershenzon, 2006), and the rate of emission was shown to be sensitive to pollinator-mediated selection (Gervasi and Schiestl, 2017; Ramos and Schiestl, 2020).

Acetic acid is a well-known microbial fermentation product, frequently emitted by microorganisms that colonized nectar (Martin et al., 2022) and regularly found in floral scents (Knudsen and Gershenzon, 2006). However, yeasts were not recorded in the nectar of *G. physocarpus* (de Vega et al., 2009); therefore, they are unlikely to be the source of the acetic acid emission. We speculate that the high amount of acetic acid emitted by *G. physocarpus* flowers falsely signals high densities of microorganisms to honeybees, thus creating the false impression that sugars have already been utilized. Volatile compounds were also detected in the nectar and can be a gustatory as well as an olfactory cue (Raguso, 2004; Burdon et al., 2020). Honeybees are able to taste acids and reject sugar solutions with added acids depending on the concentration (Frisch, 1934). It would be interesting to test whether the taste of the detected volatile compounds contributes to the avoidance of *G. physocarpus* flowers by honeybees. Perceived as olfactory cues, floral volatiles of *G. physocarpus* are not a repellent but are neutral to honeybees (Burger et al., 2017).

The floral scent of *G. physocarpus* is highly attractive for pollinating wasps. We demonstrated the attractiveness of the synthetic scent mixture only for *V. germanica* wasps, but the attractiveness of floral scent cues of *G. physocarpus* was also shown for *Polistes* and *Belanogaster* wasps in a previous study (Burger et al., 2017). Ethanol and acetic acid might be important attractants because they are commonly found in sugar-containing food sources of wasps, such as fruits (Nevo et al., 2022). Microbes that produce volatiles from the metabolism of sugars could signal suitable nutrient sources to foraging wasps (Davis et al., 2012). A high attractiveness of acetic acid for vespid wasps was already demonstrated in combination with other substances such as butyl butyrate and heptyl butyrate (Landolt, 1998). We conclude that the floral volatiles of *G. physocarpus* function as an attractant for pollinating wasps and the nectar cardenolides filter non-pollinating honeybees.

## Supplementary Material

10.1242/jexbio.246156_sup1 Supplementary information
