# Supplementary material for: Nectar cardenolides and floral volatiles mediate a specialized wasp pollination system
Source: J Exp Biol. 2024 Jan 4;227(1):jeb246156. doi: 10.1242/jeb.246156 (PMC10785657; doi:10.1242/jeb.246156)
Supplement: Supplementary information [file jexbio-227-246156-s1.pdf]

Table S1. Composition of the synthetic mixture resembling the floral volatiles of *G. physocarpus* inflorescences based on EAD-active compounds of *V. germanica* wasps. An aliquot of 50 µl corresponding to approximately 20 inflorescences was used in behavioural experiments.

**compound**	**brand**	**purity (%)**	**concentration of stock solutions (ng/µl)**	**amount of stock solution (µl)**
ethanol	Merck	100	10	16
(*Z*)-β-ocimene and (*E*)-β-ocimene	*	70	100	14
(*E*)-4,8-dimethyl-1,3,7-nonatrien	*	70	0.1	89
acetol	Aldrich		10	29
furfural	Sigma-Aldrich	99	1	83
acetic acid	Merck	99	10	157
benzaldehyde	Merck	99	10	21
linalool	Dragoco		10	153
5-methylfurfural	Aldrich	99	0.1	83
humulene	*	90	1	23
benzyl nitrile	Sigma-Aldrich		1	50
eugenol	Aldrich	99	10	25
total sum				743
*lab collection				
solvent: diethyl phtalate	Aldrich	99		

Table S2. Log Rank pairwise comparisons of the cumulative survival of honeybees in cardenolide feeding experiments (n.s.: p > 0.05, *: p < 0.05, **: p < 0.01, ***: p < 0.001, see also Fig. 5).

	G. phy high	G. phy med	G. phy low	G. fru high	G. fru low	sugar water
G. phy med	***					
G. phy low	***	***				
G. fru high	**	n.s.	***			
G. fru low	***	n.s.	n.s.	**		
sugar water	***	*	n.s.	***	n.s.	
flavonoids high	***	n.s.	**	n.s.	n.s.	*

Journal of Experimental Biology • Supplementary information
